# Phenformin Attenuates Renal Injury in Unilateral Ureteral Obstructed Mice without Affecting Immune Cell Infiltration

**DOI:** 10.3390/pharmaceutics12040301

**Published:** 2020-03-26

**Authors:** Mikkel Ø. Nørgård, Michael Christensen, Henricus A.M. Mutsaers, Rikke Nørregaard

**Affiliations:** Department of Clinical Medicine, Aarhus University, 8200 Aarhus N, Denmark; mnoergaard@health.sdu.dk (M.Ø.N.); M-christensen@live.dk (M.C.); h.a.m.mutsaers@clin.au.dk (H.A.M.M.)

**Keywords:** phenformin, metformin, renoprotective, renal inflammation

## Abstract

Phenformin and metformin are antihyperglycemic drugs that belong to the class of biguanides. Previously, we demonstrated that metformin elicits renoprotective effects in unilateral ureteral obstructed mice by reducing the infiltration of immune cells into the kidney. Since phenformin is a more potent drug as compared to metformin, we investigated the renoprotective properties of phenformin. We studied the efficacy of both drugs using mice that underwent unilateral ureteral obstruction. Renal damage was evaluated on RNA and protein level by qPCR, Western blotting, and immunohistochemistry. Moreover, we studied immune cell infiltration using flow cytometry. Both biguanides significantly reduced UUO-induced kidney injury, as illustrated by a reduction in KIM-1 protein expression. In addition, both metformin and phenformin impacted the gene expression of several inflammatory markers but to a different extent. Moreover, in contrast to metformin, phenformin did not impact immune cell infiltration into UUO kidneys. In conclusion, we demonstrated that phenformin has similar renoprotective effects as metformin, but the mechanism of action differs, and phenformin is more potent. The beneficial effects of phenformin are probably due to inhibition of the STAT3 pathway and mitochondrial complex I. Further research is needed to unveil the therapeutic potential of phenformin for the treatment of renal injury, either at low, non-toxic concentrations or as part of a combination therapy.

## 1. Introduction

Phenformin and metformin are antihyperglycemic drugs that belong to the class of biguanides; both were introduced in 1957 for the treatment of diabetes mellitus. Phenformin was widely used, while metformin—a weaker glucose-lowering biguanide—saw only limited use. However, phenformin was withdrawn from the market in the 1970s due to safety concerns associated with lactic acidosis [1]. Today, metformin is the first-choice treatment for type 2 diabetes worldwide [2]. Moreover, in the last decades, several studies have discovered additional beneficial effects of metformin treatment, such as increased survival of colorectal cancer patients with diabetes mellitus [3]. Furthermore, metformin is demonstrated to protect the kidney in various renal disease models, such as chronic kidney disease (CKD)-related vascular calcification and diabetic nephropathy [4,5,6,7]. In addition, it has been shown that metformin attenuates fibrosis during prolonged unilateral ureteral obstruction (UUO) [8,9,10] and prevents inflammation and tubular damage in mice subjected to 3 days UUO [6].

The beneficial effects of metformin in type 2 diabetes is partly attributed to organic cation transporter 1 (OCT1), which is responsible for metformin uptake by hepatocytes in the liver [7]. Compared to metformin, phenformin is less polar and more lipophilic, enabling it to pass cell membranes by passive diffusion [8].

Metformin is known to activate AMP-activated protein kinase (AMPK), which has been associated with its renoprotective effects by downregulation of transforming growth factor-β1 [11,12]. Yet, we have previously demonstrated that metformin can prevent inflammation and tubular damage in vivo independent of OCT1, and 2, as well as AMPK-β1, the most abundant AMPKβ isoform in the kidney [6]. Thus, the molecular mechanisms underlying the renoprotective effects of metformin have been partially elucidated. Yet, to fully understand the impact of metformin on renal injury, it is essential to investigate other biguanides, such as phenformin.

Phenformin has greater biological activity and anti-tumorigenic efficacy as compared to metformin [10] and is a more potent inhibitor of mitochondrial complex I [13]. Therefore, we aimed to evaluate whether phenformin has greater renoprotective effects than metformin, which has not been studied to date, and to further explore the potential mechanisms driving the renoprotective effects of biguanides, which will aid future drug development.

## 2. Methods

### 2.1. Housing and Biguanide Treatment

All animal experiments were performed in cooperation with a veterinarian in accordance with the Danish National Guidelines for the care and handling of experimental animals. All experimental protocols were approved by the Department of Clinical Medicine, Aarhus University, according to the licenses for the use of experimental animals issued by the Animal Experiments Inspectorate, under the Danish Veterinary and Food Administration (no. 2015-15-0201-00658; 3 September, 2015).

Seven-week-old male C57BL/6NRj mice (Janiver Labs, Le Genest-Saint-Isle, France) had ad libitum access to standard rodent diet (Altromin, Lage, Germany) and tap water. They were housed at a temperature of 21 ± 2 °C with a 12 h:12 h light-dark cycle and a humidity of 55 ± 5%. Mice were randomly divided into each experimental group: Sham (*n* = 15, six kidneys were prepared for quantitative PCR (qPCR) and western blotting (WB); 4 kidneys were prepared for immunohistochemistry [IHC], and five kidneys were used for Flow cytometry) and unilateral ureteral obstruction (UUO) with vehicle treatment as well as UUO with metformin or phenformin (*n* = 17 for each group; 8 kidneys were prepared for qPCR and WB; four kidneys were prepared for IHC, and five kidneys were used for Flow cytometry) Metformin (Sigma-Aldrich, St. Louis, MO, USA) was administered via the drinking water (500 mg/kg/day) and phenformin was administrated through daily gavage (100 mg/kg/day). The concentrations were estimated on an average weight measurement every second day. Based on previous measurements, mice were assumed to drink 4.5 mL of water per day. Phenformin was dissolved in 200 µL of water and applied by gavage. The mice received treatment starting seven days prior to the obstruction until they were sacrificed.

### 2.2. Unilateral Ureteral Obstruction

Mice were anesthetized with 2% sevoflurane (Abbot Scandinavia, Solna, Sweden) mixed with O_2_ (2 L/min) during surgery, and injected with Temgesic (Reckitt Berkshire, Slough, UK) directly thereafter. During the procedures, animals were placed on a heating pad to maintain physiological body temperature. The abdomen was shaved and cleaned with ethanol, and an incision was made to expose the left ureter, which was subsequently obstructed by tying a silk ligature around it. The mice received Temgesic in their drinking water for 48 h post-surgery. Sham-operated mice underwent the same procedure without being obstructed. At the end of the experiment, blood was collected via cardiac puncture. Immediately afterward, the left kidney was removed and either frozen or used immediately depending on the analysis methods.

Blood samples were centrifuged, and plasma levels of sodium, potassium, and urea were measured using a Roche Cobas 6000 analyzer (Roche Diagnostic). Creatinine levels were quantified using the Creatinine Assay Kit (Sigma) according to the manufacturer’s instructions.

### 2.3. Cell Preparation and Flow Cytometry

Right after removal of the left kidney, half a kidney was dissociated using the Multi Tissue Dissociation Kit 2 (Miltenyi Biotec, Bergisch Gladbach, Germany) and the gentleMACS Octo Dissociator (Miltenyi Biotec). The obtained cell suspension was subsequently passed through a cell strainer (100 µm) and washed in washing buffer (PBS pH 7.4 containing 0.5% bovine serum albumin [BSA] and 0.09% NaN_3_). Afterward, the cell suspension was incubated for 15 min with a premade antibody (V450-CD11b, BD Biosciences, San Jose, CA, USA, FITC-Ly6C, Miltenyi Biotec, Germany and BioLegend, USA, Alexa Flour 647-F4/80, Biolegend, San Diego, CA, USA), including Near IR-Live/Dead Fix (Thermo Fisher Scientific). Next, the samples were incubated with BD FACS Lysing Solution (BD Biosciences, San Jose, CA, USA), washed and resuspended in phosphate-buffered saline (PBS) before being analyzed on a BD LSRFortessa (BD Biosciences) using BD FACSDiva™software v8. Data were analyzed using FlowJo 10 (TreeStar, Ashland, OR, USA). All data were time-gated to exclude any abnormalities, as well as doublets and dead cells. The SHAM, UUO, and metformin groups have previously been used in a different study [14], but all groups, including the phenformin group, were analyzed at the same time.

### 2.4. Western Blot

Renal cortex was homogenized in a dissociation buffer (0.3 sucrose, 25 mM imidazole, 1 mM EDTA, pH 7.2) including phosphatase-inhibitor 2 and 3 (Sigma Aldrich, St. Louis, USA) and a mini protease inhibitor cocktail tablet (Roche Diagnostics, Hvidovre, Denmark) using a tissuelyser RT (Qiagen, Hilden, Germany), followed by centrifugation. The total protein content of the supernatant was determined using the Pierce BCA Protein Assay Kit (Thermo Scientific, Roskilde, Denmark).

2% SDS was added to the supernatants to prepare gel samples. Protein samples (20 µg) were separated on a Criterion TGX Stain-free gel (BioRad, Denmark) and subsequently transferred to a nitrocellulose membrane (Bio-Rad, Copenhagen, Denmark). The membranes were blocked with 5% skimmed milk in TBS-Tween and incubated with the primary antibodies overnight at 4 °C. Membranes were washed again and incubated with the secondary antibodies for 1 h at room temperature. Proteins were visualized by a chemiluminescence system (Bio-Rad ChemiDocTM Imager). Proteins of interest were normalized to total protein content measured with stain-free technology [11]. This stain-free technology has several advantages; 1) assures loading of proteins to the gel, 2) verifies the equal transfer of protein from the gel to the nitrocellulose membrane and 3) allows normalization to total protein, which is measured on the same membrane as used for detection of the protein of interest. Primary and secondary antibodies are listed in Table 1.

### 2.5. Quantitative PCR

RNA was extracted from renal cortical tissue using the Nucleospin RNA II mini kit (Macherey Nagel, Düren, Germany) according to the manufacture’s protocol. Afterward, RNA concentration was determined by spectrophotometry at 260 nm. cDNA was synthesized using the AffinityScript qPCR cDNA synthesis kit (Life Technologies, Thermo Fischer Scientific, Waltham, MA, USA). qPCR was performed using the SYBR^®^ Green qPCR Master Mix and run with on an Aria MX3000p (Agilent Technologies, Lexington, MA, USA). Primers are listed in Table 2. Two reference genes were included in each PCR run; data normalization was performed with the reference gene that showed the least amount of regulation to prevent misinterpretation of the gene expression profile.

### 2.6. Histology

Kidneys were fixed with 4% paraformaldehyde in a 0.01 M PBS buffer by retrograde perfusion via the left ventricle. Subsequently, the kidneys were fixed for an additional hour. Afterwards, the tissue was washed in PBS buffer, dehydrated in graded ethanol and left overnight in xylene. The tissue was embedded in paraffin and sectioned using a rotary microtome (Thermo Scientific, Microm HM 355S). 2 µm sections were stained with hematoxylin and eosin (H&E) to visualize morphology and tubular damage.

### 2.7. Immunofluorescence Staining

For immunofluorescence staining, the sections were deparaffinized in xylene, rehydrated in graded ethanol (99–70%), and boiled in TEG-buffer to improve target availability. To prevent non-specific binding, tissue sections were blocked with 50 mM NH_4_Cl in PBS and subsequently incubated in PBS with 1% BSA, 0.2% gelatin and 0.05% saponin. Next, slides were incubated overnight with KIM-1 antibody (R&D Systems) diluted in PBS containing 0.1% BSA and 0.3% Triton X-100 at 4 °C. After incubation, slides were washed with PBS containing 0.1% BSA, 0.2% gelatin and 0.05% saponin, and subsequently incubated with a secondary antibody (Alexa Fluor 568) and counterstained with DAPI (Sigma). Slides were mounted using Slowfade Light Antifade (Invitrogen, Carlsbad, CA, USA). Sequential overlapping images were taken and merged in Photoshop© CS5.

In order to evaluate fluorescence intensity, 10 pictures (20× magnification) distributed throughout the whole kidney were used. The threshold was set using a positive and negative control, and ImageJ software was used to calculate the intensity of KIM-1 immunolabeling.

### 2.8. Statistical Analysis

Statistical analysis was performed using GraphPad Prism v7 (GraphPad Software Inc., San Diego, CA, USA). Data are presented as mean ± SEM. All data were analyzed using a one-way ANOVA followed by Tukey’s multiple-comparison test. If data was not normally distributed, it was analyzed using a Kruskal–Wallis test followed by Dunn’s multiple comparison test. *p*-values < 0.05 were considered significant.

## 3. Results

### 3.1. Phenformin Induces Elevated Blood Lactate Levels in UUO Mice

As expected, mice subjected to 3dUUO had an increased kidney weight (KW) as compared to sham mice. Metformin treatment (500 mg/kg/day) further increased KW, while phenformin treatment (100 mg/kg/day) did not significantly affect KW as compared to untreated UUO mice (Table 3). In addition, treatment with phenformin (100 mg/kg/day) significantly decreased blood pH and glucose, and increased blood lactate levels as compared to UUO mice, which might be associated with lactic acidosis. Nonetheless, all mice displayed normal intake of water and food and were considered to be in good condition by our animal caretakers.

### 3.2. Biguanides Attenuate Tubular Injury in Response to 3dUUO

**Figure 1:**
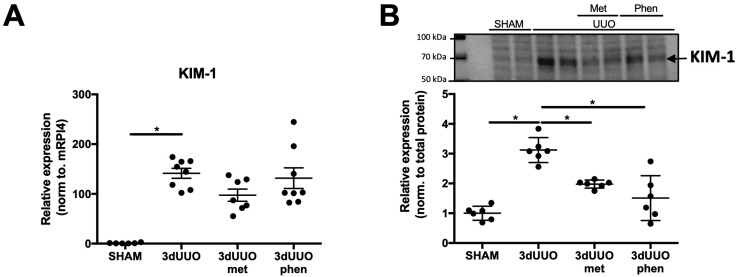
**Phenformin attenuates the expression of KIM-1 following UUO in a similar fashion as metformin.** (**A**) Relative mRNA expression of kidney injury molecule-1 (KIM-1) corrected for ribosomal protein L4 (RPl4), *n* = 6–8 for each group. (**B**) KIM-1 protein expression normalized to total protein, *n* = 6 for each group. Each bar represents the mean ± SEM. * *p* < 0.05.

**Figure 2:**
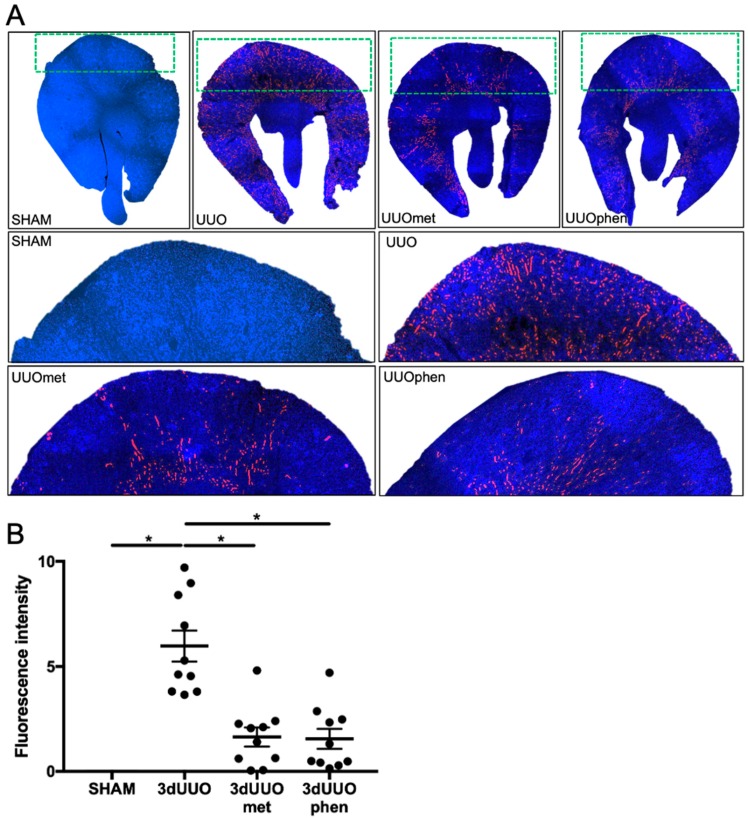
**Protein expression of KIM-1 following UUO and biguanide treatment.** (**A**) Whole kidneys and enlarged take-outs stained for KIM-1 (red) and nuclei (blue). 10 pictures were captured for each kidney in order to cover the whole cortex, *n* = 4 for each group. (**B**) Quantification of KIM-1 staining. Each bar represents the mean ± SEM. * *p* < 0.05.

**Figure 3:**
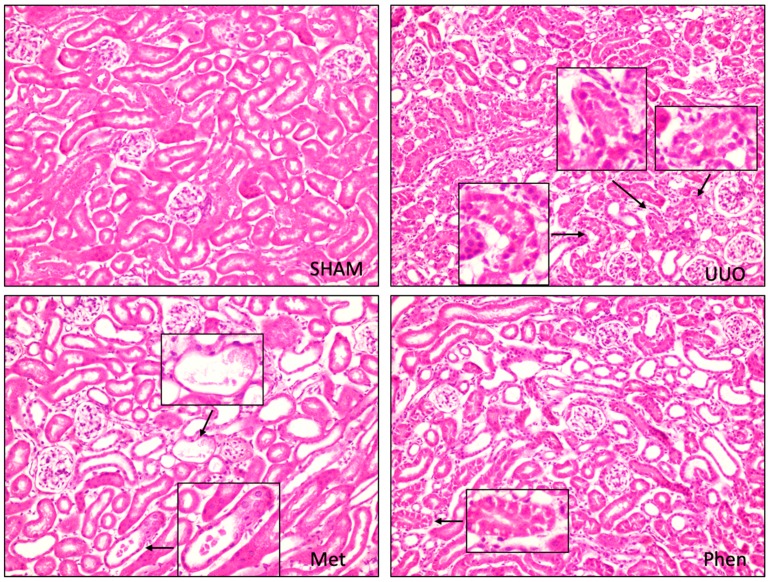
**Representative images of renal morphology.** Injured tubules are marked by arrows. 20× magnification, *n* = 4 for each group.

Expression of kidney injury molecule-1 (KIM-1)—a biomarker for renal proximal tubule injury [12]—increased in UUO mice as compared to sham-operated mice (Figure 1). Both phenformin and metformin did not significantly reduce UUO-induced KIM-1 mRNA expression (Figure 1A), however, a significant reduction was observed on protein level (Figure 1B). In order to evaluate the cortical localization of KIM-1, fluorescence microscopy was performed (Figure 2A). UUO surgery resulted in a more than five-fold increase in staining intensity in the kidneys, which was reduced by treatment with both biguanides (Figure 2B). Additionally, Hematoxylin and Eosin (H&E) staining was performed, which suggested a reduction in tubular damage following biguanide treatment as compared to non-treated animals (Figure 3).

### 3.3. Effects of Biguanides on Oxidative Stress in Response to 3dUUO

**Figure 4:**
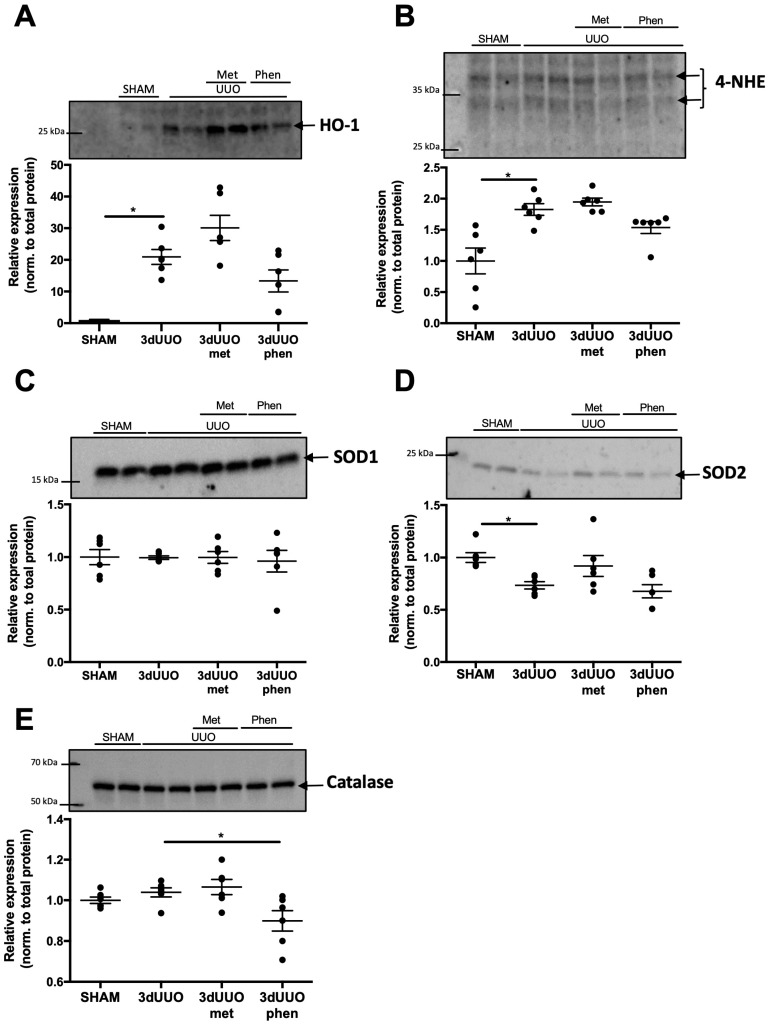
**Regulation of oxidative stress markers and antioxidants following UUO and biguanide treatment.** (**A–E**) Protein expression of the oxidative stress markers Hemeoxygenase-1 (HO-1), 4-Hydroxynonenal (4-HNE), Superoxide dismutase 1 and 2 (SOD1, 2), and Catalase in response to UUO and biguanide treatment normalized to total protein. *n* = 6 for each group. Each bar represents the mean ± SEM. * *p* < 0.05.

Reactive oxygen species (ROS) are an integral part of UUO-induced fibrogenesis [15]. Therefore, we investigated whether metformin and phenformin affected oxidative stress markers. Hemeoxygenase-1 (HO-1) and 4-Hydroxynonenal (4-HNE) were upregulated in mice subjected to 3dUUO (Figure 4A,B), but no significant difference was observed following treatment with phenformin or metformin. Superoxide dismutase 1 and 2 (SOD1, 2) and catalase are antioxidants responsible for the reduction of ROS. Protein expression of SOD1 was not affected by either UUO or biguanide treatment, whereas the SOD2 protein level was reduced in response to UUO but not affected by treatment (Figure 4C,D). Protein expression of catalase was not affected by UUO or metformin, but phenformin significantly reduced catalase levels (Figure 4E). Although no significant differences were found, the expression of oxidative stress markers was generally higher in UUO mice treated with metformin compared to phenformin-treated UUO mice (Figure 4A,B,D,E).

### 3.4. Biguanides Differentially Impact the Inflammatory Response in UUO Mice

**Figure 5:**
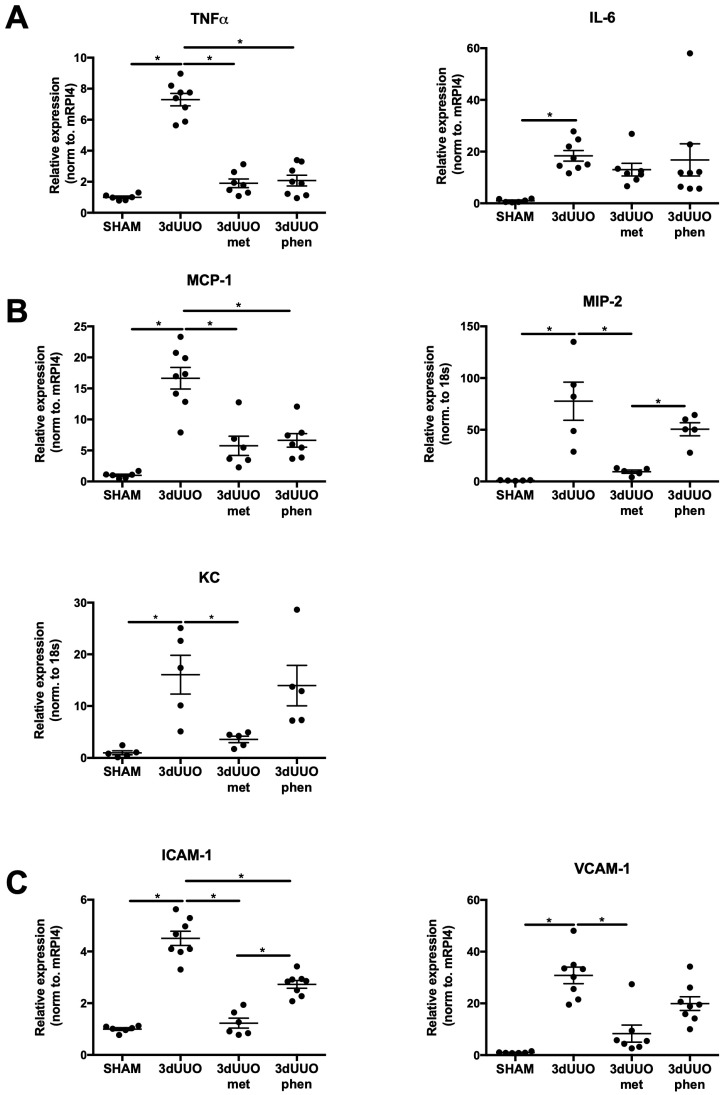
**Biguanides differentially impact cytokine expression in response to UUO.** (**A**) Relative mRNA expression of the inflammatory markers tumor necrosis factor α (TNFα) and interleukin 6 (IL-6) in response to 3dUUO and biguanide treatment normalized to ribosomal protein L4 (RPl4). (**B**) Relative mRNA expression of macrophage inflammatory protein 2 (MIP-2) and keratinocyte-derived chemokine (KC) in response to UUO and biguanide treatment normalized to 18s. (**C**) Relative mRNA expression of intercellular adhesion molecule 1 (ICAM-1) and vascular cell adhesion 1 (VCAM-1) mRNA in response to UUO and biguanide treatment normalized to RPl4. *n* = 6–8 for each group. Each bar represents the mean ± SEM. * *p* < 0.05.

To investigate the effect of both drugs on renal inflammation we measured the mRNA expression of the cytokines: tumor necrosis factor α (TNFα) and interleukin 6 (IL-6), the chemokines: monocyte chemoattachment protein-1 (MCP-1), macrophage inflammatory protein 2 (MIP-2), and keratinocyte-derived chemokine (KC) as well as the adhesion molecules: intercellular adhesion molecule 1 (ICAM-1) and vascular cell adhesion 1 (VCAM-1). Mice subjected to 3dUUO demonstrated increased levels of all markers (Figure 5). Both metformin and phenformin attenuated UUO-induced mRNA expression of TNFα, MCP-1, and ICAM-1 (Figure 5A–C). In contrast, the gene levels of MIP-2, KC, and VCAM-1 were only significantly reduced by metformin treatment and not by phenformin (Figure 5B,C). In addition, IL-6 expression was not affected by either treatment (Figure 5A).

### 3.5. Biguanides Affect Protein Expression of the pSTAT3 Pathway

**Figure 6:**
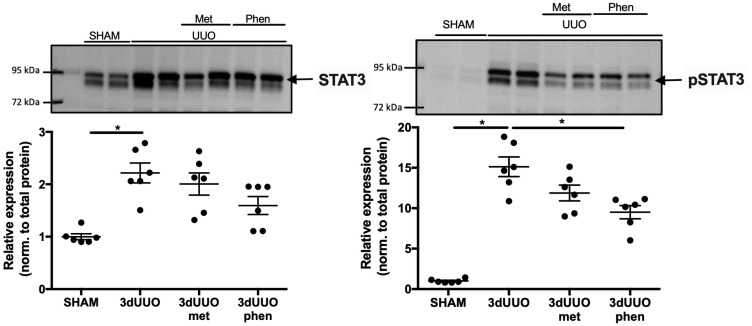
**Impact of biguanide treatment on pSTAT3 protein expression following UUO.** Protein expression of Signal transducer and activator of transcription 3 (STAT3) and phosphorylated STAT3 (pSTAT3) in response to UUO and biguanide treatment normalized to total protein. *n* = 6 for each group. Each bar represents the mean ± SEM. * *p* < 0.05.

Signal transducer and activator of transcription 3 (STAT3) and phosphorylated STAT3 (pSTAT3) play a regulatory role in cellular differentiation and are involved in the maturation of monocytes [16]. Both total STAT3 and pSTAT3 were increased in response to 3dUUO, while only phenformin attenuated UUO-induced pSTAT3 expression (Figure 6).

### 3.6. Impact of Metformin and Phenformin on Immune Cell Infiltration

We previously demonstrated that metformin reduces the number of immune cells in UUO kidneys [14]. Here we investigated whether phenformin had a similar effect. 

**Figure 7:**
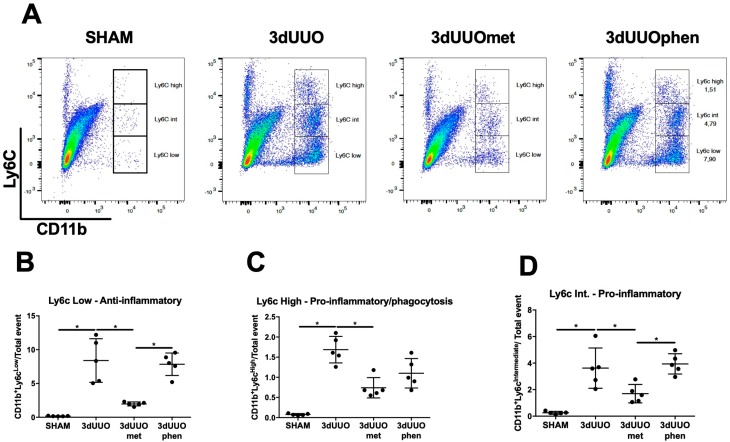
**Ly6C monocyte cell infiltration into the UUO kidney is not affected by phenformin.** (**A**) Dot-plot of kidney cells separated by cluster of differentiation 11b (CD11b) and Lymphocyte antigen 6 complex (Ly6C) expression. (**B**–**D**) Graphs showing the relative proportion of monocyte subpopulations in the kidney defined by Ly6C expression. *n* = 5 for each group. Each bar represents the mean ± SEM. * *p* < 0.05.

Expression of cluster of differentiation 11b+ (CD11b+) and Lymphocyte antigen 6 complex (Ly6C) can be used to identify subpopulations of monocyte/macrophages in numerous tissues, including the kidney [6,17]. The infiltration and activation of different leukocytes in the kidney is a hallmark of chronic kidney disease [18], and specific subpopulations of Ly6C monocytes are recruited to the kidney during UUO [19]. All monocyte subpopulations were increased in UUO kidneys, which were reduced by metformin treatment (Figure 7). Conversely, phenformin did not significantly affect monocyte infiltration (Figure 7).

**Figure 8:**
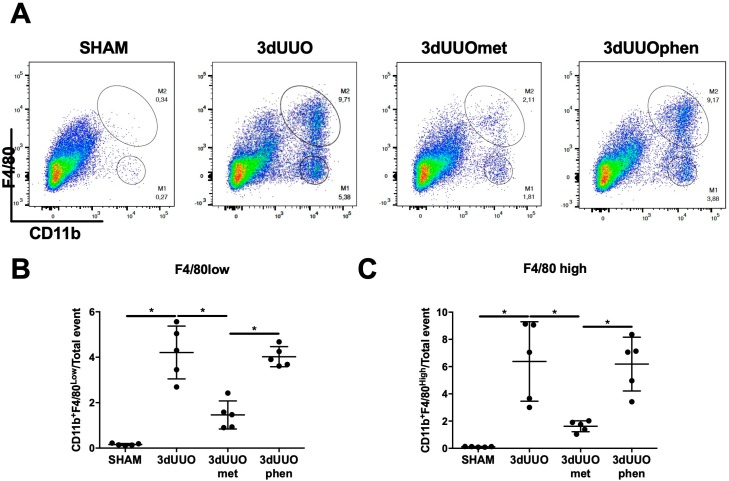
**M1 and M2 cell infiltration into the UUO kidney is not affected by phenformin.** (**A**) Dot-plot of kidney cells separated by the cluster of differentiation 11b (CD11b) and EGF-like module-containing mucin-like hormone receptor-like 1 (F4/80) expression, identifying M1 and M2 monocytes/macrophages. (**B**,**C**) Graphs of the proportions of M1 and M2 monocytes defined by F4/80 expression. *n* = 5 for each group. Each bar represents the mean ± SEM. * *p* < 0.05.

The surface markers CD11b+ and EGF-like module-containing mucin-like hormone receptor-like 1 (F4/80) can be used to distinguish between different macrophage subtypes, namely pro-inflammatory M1 and anti-inflammatory M2 macrophages [20]. Both F4/80low and F4/80high cells were increased in UUO kidneys (Figure 8). Metformin treatment significantly decreased the percentage of these cells in UUO kidneys, while phenformin did not have any effect (Figure 8).

## 4. Discussion

The main result of this study is that phenformin attenuates tubular damage and inflammation to a similar degree as metformin in UUO mice, albeit at a lower dose. However, in contrast to metformin, phenformin did not affect the renal infiltration of monocytes and macrophages. The renoprotective effects of metformin described here are consistent with previous findings [6], but, to the best of our knowledge, this is the first in vivo study demonstrating the renoprotective effects of phenformin.

To elucidate whether the renoprotective effect of phenformin acts through similar pathways as metformin, we tested the effect of phenformin on several markers of renal inflammation. Our results demonstrated that phenformin lowered the expression of multiple inflammatory markers, similar to metformin; however, we did not observe an immunoregulatory effect of phenformin, in contrast to metformin. Following UUO, the expression of VCAM and ICAM was shown to be increased. These proteins play an important role in chemotaxis and adhesion of leukocytes to the site of injury [21]. We have recently shown that metformin decreases the expression of VCAM and ICAM and limits immune cell infiltration into UUO kidneys [6]. In addition, metformin modulates immune cell composition on a systemic level, indicating whole-body mechanisms underlying the renoprotective effects of metformin [14]. However, our current data indicate that the renoprotective effects of phenformin are independent of immunoregulatory effects. When analyzing specific subpopulations of monocytes recruited to renal tissue during UUO, we found an increase in all the different subpopulations expressing Ly6C, as observed previously by others [19]. In contrast to metformin, phenformin did not reduce the number of infiltrating Ly6C cells. When looking at monocyte subpopulations, based on the M1/M2 classification, we observed a similar pattern. Both M1/M2 macrophage subtypes were increased in the UUO kidney, which was mitigated by metformin treatment but not by phenformin treatment. Taken together, our results demonstrated that phenformin has renoprotective effects comparable to those of metformin, but the mechanism of action of both biguanides is different.

The anti-oncogene effects of metformin have been suggested to involve inhibition of the STAT3 pathway, which can also prevent monocyte-to-macrophage differentiation [16,22]. We previously postulated that metformin attenuated immune cell infiltration by reducing pSTAT3 expression [14]. However, we did not observe any immunoregulatory effects of phenformin, despite a significant impact on pSTAT3 levels. It has been shown that STAT3 plays a key role in the communication between damaged renal tubular cells and surrounding fibroblasts. Activation of STAT3 in tubular cells promotes the activation of fibroblasts/pericytes, which ultimately leads to interstitial fibrosis [23]. Moreover, it has been demonstrated that STAT3-deficient fibroblasts are less sensitive to the pro-fibrotic effects of TGF-β, and fibroblast-specific knockout of STAT3 attenuates skin fibrosis in experimental mouse models [24]. Taken together, the renoprotective effects of biguanides most likely result from a reduction in STAT3 activation; however, the immunoregulatory effects of metformin are probably independent of the STAT3 pathway.

Oxidative stress plays an important role in the pathogenesis of UUO [6,25]. Mitochondrial complex I and III are important sources of ROS in the kidney [26]. We have previously shown that rotenone, a complex I inhibitor, attenuates UUO-induced oxidative stress [27]. Since phenformin is also capable of inhibiting complex I, [13,28] we hypothesized that the renoprotective effects of phenformin could involve a reduction in oxidative stress. Our results showed that phenformin reduced the induction of several cytoprotective enzymes such as HO-1 and catalase in UUO mice; this suggests that phenformin reduced oxidative stress. However, more research is needed to fully elucidate the oxidative state of UUO mice following phenformin treatment.

Lactic acidosis is a serious and potentially lethal complication that can arise from phenformin treatment, which limits the clinical use of this drug. Due to these limitations, the renoprotective characteristics of phenformin have never been evaluated, and a safe dosing-regime has never been established. Here, we demonstrated that phenformin reduced renal injury at a much lower dose as compared to metformin, 100 mg/kg/day vs. 500 mg/kg/day, respectively. Therefore, future studies should be aimed at finding the lowest effective dose of phenformin as a renoprotective agent. It might be that the beneficial effects of phenformin are also present at non-toxic concentrations, as is seen in the field of cancer research [29]. Furthermore, it is worthwhile to investigate the potential synergistic effects of low doses of phenformin with other renoprotective agents such as butaprost or nintedanib [30,31].

In conclusion, the present study demonstrated that phenformin has similar renoprotective effects as metformin, but the mechanism of action differs, and phenformin is more potent. The beneficial effects of phenformin might be associated with effective inhibition of the STAT3 pathway as well as mitochondrial complex I, but further research is needed to unveil the therapeutic potential of phenformin for the treatment of renal injury, either at low, non-toxic concentrations or as part of a combination therapy.

## Figures and Tables

**Table 1:** Primary and secondary antibodies.

Target Protein	Company	Catalog #	Dilution
**Primary Antibodies**			
KIM-1	R&D systems, USA	AF1817	1:1000
STAT3	Cell Signalling, NL	mAB9139	1:1000
pSTAT3	Cell Signalling, NL	mAB4113	1:1000
HO-1	ENZO, Denmark	ADI-SPA-896-D	1:500
4-HNE	Abcam, USA	Ab46545	1:250
SOD1	ENZO, Denmark	ADI-SOD-100-D	1:1000
SOD2	ENZO, Denmark	06-984	1:1000
Catalase	Abcam, USA	Ab76024	1:1000
**Secondary Antibodies**			
Goat anti-mouse immunoglobulin/HRP	DAKO, Denmark	P0447	
Goat anti-rabbit immunoglobulin/HRP	DAKO, Denmark	P0448	
Rabbit anti-goat immunoglobulin/HRP	DAKO, Denmark	P0449	

**Table 2:** qPCR primers.

Target Gene	Direction	Sequence
KIM-1	Forward Reverse	5′-CGGTACAACTTAAAGGGGCA-3′ 5′-GACGTGTGGGAATCTCTGGT-3′
MCP-1	Forward Reverse	5′-CAAGAAGGAATGGGTCCAGA-3′ 5′-GTGCTGAAGACCTTAGGGCA-3′
ICAM-1	Forward Reverse	5′-TCCAATTCACACTGAATGCC-3′ 5′-GTCTGCTGAGACCCCTCTTG-3′
VCAM-1	Forward Reverse	5′-GTGGTGCTGTGACAATGACC-3′ 5′-ACGTCAGAACAACCGAATCC-3′
MIP-2	Forward Reverse	5′-CTCTCAAGGGCGGTCAAAAAGTT-3′ 5′-TCAGACAGCGAGGCACATCAGGTA-3′
KC	Forward Reverse	5′-GCGAATTCACCATGATCCCAGCCACCCG-3′ 5′-GCTCTAGATTACTTGGGGACACCTTTTAG-3′
TNF-α	Forward Reverse	5′-AGGCTGCCCCGACTACGT-3′ 5′-GACTTTCTCCTGGTATGAGATAGCAAA-3′
IL-6	Forward Reverse	5′-GATGCTACCAAACTGGATATAATC-3′ 5′-GGTCCTTAGCCACTCCTTCTGTG-3′
RPl4	Forward Reverse	5′-CTTTGCCAGCTGTGTTCAA-3′ 5′-ATTTCACTGACGGCATAGGG-3′
18S	Forward Reverse	5′-TGTGGTGTTGAGGAAAGCAG-3′ 5′-TCCCATCCTTCACATCCTTC-3′

**Table 3:** Physiological parameters.

Parameter	SHAM (*n* = 6)	3dUUO (*n* = 8)	3dUUOmet (*n* = 8)	3dUUOphen (*n* = 8)
Weight (g)	21.7 ± 1.02	21.28 ± 0.36	21.72 ± 0.27	21.78 ± 0.35
KW (mg/25g mouse)	156.5 ± 6.17	235.35 ± 4.19 ^A^	279.37 ± 6 ^AB^	244.99 ± 6.39 ^AC^
pH	7.25 ± 0.01	7.25 ± 0.02	7.24 ± 0.01	7.14 ± 0.02 ^ABC^
Glucose (mmol/L)	8.42 ± 0.44	8.14 ± 0.33	8 ± 0.36	5.04 ± 0.0 ^ABC^
Lactate (mmol/L)	6.18 ± 0.35	7.10 ± 0.60	7.59 ± 0.17	10.91 ± 0.0 ^ABC^
Creatinine (μmol/L)	14.89 ± 0.83	13.60 ± 1.60	14.32 ± 0.45	14.16 ± 1.21
Na^+^ (mmol/L)	148.40 ± 0.46	150.50 ± 0.43 ^A^	150.71 ± 0.33 ^A^	151.63 ± 0.35 ^A^
K^+^ (mmol/L)	4.64 ± 0.12	5.00 ± 0.12	4.70 ± 0.05	5.00 ± 0.13
BUN (mmol/L)	7.32 ± 0.70	9.23 ± 0.44	9.00 ± 0.39	8.14 ± 0.40

A denotes *p* < 0.05 compared with SHAM, B denotes *p* < 0.05 compared with UUO, C denotes *p* <0.05 compared with 3dUUOmet.
